# Proteomic Dynamics of Multidrug Resistance Mechanisms in Lucena 1 Cell Line

**DOI:** 10.3390/cells13171427

**Published:** 2024-08-26

**Authors:** Emidio Beraldo-Neto, Fernanda Cardoso Amador, Karolina Rosa Fernandes, Giselle Zenker Justo, José Thalles Lacerda, Maria A. Juliano

**Affiliations:** 1 Biochemistry Laboratory, Butantan Institute, São Paulo 05503-900, Brazil; 2 Department of Biophysics, Paulista School of Medicine, Federal University of São Paulo, São Paulo 04023-062, Brazil; 3 Department of Biochemistry, Paulista School of Medicine, Federal University of São Paulo, São Paulo 04023-062, Brazil

**Keywords:** Lucena 1, K562 cell line, multi drug resistance, P-glycoprotein, Leukemia

## Abstract

The Lucena 1 cell line, derived from the human chronic myeloid leukemia cell line K562 under selective pressure of vincristine supplementation, exhibits multidrug resistance (MDR). This study aims to explore and elucidate the underlying mechanisms driving MDR in the Lucena 1 cell line. A proteomic analysis comparing K562 and Lucena 1 revealed qualitative differences, with a focus on the ATP-dependent efflux pump, Translocase ABCB1, a key contributor to drug resistance. Tubulin analysis identified two unique isoforms, Tubulin beta 8B and alpha chain-like 3, exclusive to Lucena 1, potentially influencing resistance mechanisms. Additionally, the association of Rap1A and Krit1 in cytoskeletal regulation and the presence of STAT1, linked to the urea cycle and tumor development, offered insights into Lucena 1’s distinctive biology. The increased expression of carbonic anhydrase I suggested a role in pH regulation. The discovery of COP9, a tumor suppressor targeting p53, further highlighted the Lucena 1 complex molecular landscape. This study offers new insights into the MDR phenotype and its multifactorial consequences in cellular pathways. Thus, unraveling the mechanisms of MDR holds promise for innovating cancer models and antitumor targeted strategies, since inhibiting the P-glycoprotein (P-gp)/ABCB1 protein is not always an effective approach given the associated treatment toxicity.

## 1. Introduction

Leukemia is a hematological malignancy blood cancer that arises from the clonal proliferation of hematopoietic stem cells (HSCs) in the bone marrow and peripheral blood [1,2]. The uncontrolled growth and accumulation of abnormal white blood cells characterize the disease, interfering with the normal production and function of erythrocytes, platelets, and other immune cells. This results in many symptoms and complications, including anemia, bleeding, and organ failure [3,4].

It has several classifications exhibiting distinct genetic, molecular, and clinical features. Acute lymphoblastic leukemia (ALL) is a rapidly progressing disease that predominantly affects children, whereas chronic lymphocytic leukemia (CLL) is a slow-growing disease that mainly affects adults [5]. Acute myeloid leukemia (AML) is a highly aggressive disease affecting adults and children. In contrast, chronic myeloid leukemia [3] (CML) is a slowly progressing disease associated with a unique genetic abnormality known as the Philadelphia chromosome.

The balance between self-renewal and differentiation of HSCs is perturbed in leukemia, accumulating immature and dysfunctional blood cells. The pathogenesis of leukemia involves a complex interplay between genetic, epigenetic, and microenvironmental factors that perturb the balance between self-renewal and differentiation of HSCs [6]. Acquiring genetic mutations and chromosomal abnormalities, such as translocations, deletions, and amplifications, can drive the leukemic transformation of HSCs and alter their differentiation potential [7].

K562, K562-Lucena 1 are commonly used cell lines for studying leukemia, and they share some similarities [8]. K562 is a human chronic myeloid leukemia (CML) cell line isolated in 1972. It is commonly used as a model for studying erythroid and myeloid differentiation and for drug screening and discovery. K562 cells are able to differentiate into erythroid-like cells in response to various stimuli. K562-Lucena 1 (also known as Lucena 1) is a modified subline of K562 cells generated by stepwise selection in vincristine to overexpress the P-gp/ABCB1 [9], whose activity and expression levels are considered an independent risk factor for treatment and drug resistance [10,11,12,13,14].

Resistance to multiple chemotherapeutic drugs, known as the multidrug resistance (MDR) phenotype, is one aspect of treatment failure and cancer progression, and it is frequently associated with the overexpression of the ABC transporter proteins [15,16]. The P-gp/ABCB1 transporter (also known as MDR1) is a transmembrane efflux pump that uses ATP to actively transport substances, differing in both structure and function, out of the cell against their concentration gradients [17]. The development of multidrug resistance is a significant challenge in treating leukemia and other cancers [10,17]. Thus, understanding the molecular and cellular mechanisms underlying drug resistance in MDR leukemia cell lines is essential for developing effective treatment strategies for patients with drug-resistant leukemia. The aim of this study was to explore and elucidate the mechanisms driving the MDR phenotype in the Lucena 1 cell line and its multifactorial consequences in cellular pathways, using proteomic analysis.

## 2. Methods

### 2.1. Cell Lines and Culture Conditions

K562 cells were purchased from the American Type Culture Collection (ATCC, Rockville, MD, USA), and the resistant cell line Lucena 1 was kindly donated by Prof. Vivian Rumjanek (Federal University of Rio de Janeiro, Brazil). Lucena 1 cells derived from K562 cells as previously described [9]). K562 and Lucena 1 cells were routinely maintained at 37 °C in RPMI 1640 medium (GIBCO, Carlsbad, CA, USA) supplemented with 2 mM glutamine (SIGMA, Darmstadt, Germany), 100 U/mL penicillin (Gibco), 100 ug/mL streptomycin (GIBCO, Carlsbad, CA, USA), and 10% fetal bovine serum (GIBCO, Carlsbad, CA, USA) in a 5% CO_2_ humidified atmosphere. Lucena 1 cells were grown in the presence of 60 nM vincristine (VCR; SIGMA-ALDRICH, St. Louis, MO, USA) in the culture medium [9]. Cellular viability was determined by the tripan blue exclusion assay.

For experiments, K562 and Lucena 1 cells (100,000 cells/mL) were grown in 25 cm^2^ culture flasks for 24 h in serum-free RPMI 1640 medium supplemented with glutamine and antibiotics at 37 °C in a 5% CO_2_ humidified atmosphere. Lucena 1 cells were grown in the presence of 60 nM vincristine in the culture medium [9].

### 2.2. Sample Preparation and In-Solution Digestion

The cell experiments were conducted in triplicate (*n* = 3 culture flasks), and samples were centrifuged at 3000 rpm for 10 min. Centrifugation resulted in the cell pellet, which was then resuspended in PBS buffer and centrifuged three additional times. In the third centrifugation, the pellet was resuspended in ultrapure water, and pooled samples were prepared. The pooled samples were sonicated using a sonicator for 60 s.

The sonication-homogenized samples were quantified by spectrophotometer readings (at 280 nm) and standardized for their protein content for future comparative analysis. Subsequently, they underwent solution digestion, and 20 µL of 50 mM ammonium bicarbonate was added, and the sample was reduced by adding 2 µL of 100 mM DTT (SIGMA-ALDRICH, St. Louis, MO, USA) at 60 °C for 30 min. Then, samples were alkylated by adding 2 µL of 200 mM iodoacetamide (SIGMA-ALDRICH, St. Louis, MO, USA) at room temperature for 30 min. Reaction was kept protected from light. Samples were digested by trypsin (1 µg, Trypsin Singles, Proteomics Grade, SIGMA-ALDRICH, St. Louis, MO, USA) overnight at 37 °C. The reaction was stopped with 5 µL of acetic acid.

### 2.3. Proteomic Analysis

All digested samples were then analyzed in technical duplicates; for proteomic analysis, samples were cleaned up using C18 ZipTips (© 2023 Merck KGaA, Darmstadt, Germany). One microliter of the tryptic peptides was subjected to nano-ESI-LC-MS/MS using a Dionex Ultimate 3000 RSLCnano (Thermo Fisher Scientific, Waltham, MA, USA) coupled with an Impact II mass spectrometer (Bruker Daltonics, Bremen, Germany). Fractions were injected in a nano-trap Acclaim PepMap (Dionex-C18, 100 Å, 75 μm × 2 cm) in 2% solvent A2 (0.1% formic acid) for 2 min under a 5 µL·min^−1^ flow rate. Elution was performed by a linear gradient of 5–40% of solvent B2 (0.1% formic acid in acetonitrile) in 120 min, under 350 nL min^−1^. Mass spectra were acquired in positive mode. MS and MS/MS scans were acquired at 2 Hz, in a *m*/*z* 50–2000 range. CID energy ramped between 7 and 70 eV. Data were processed by Peaks Studio version 8.5 (Bioinformatics Solution Inc., Waterloo, ON, Canada), and data were searched against Human Reference Proteome (UP000005640) from UniProt.

## 3. Results

In the proteomic analysis aimed at identifying differential factors in the Lucena 1 cell line, we conducted a comparative analysis to select its unique proteins. A total of 3399 proteins and 15,887 peptide sequences were identified for the Lucena 1, while 2686 proteins and 13,391 peptide sequences were identified for the K562 group. To enhance the accuracy of protein selection, we applied a logarithmic cut-off of −10lgP > 90, resulting in the final identification of 36 unique proteins for the Lucena 1 group, as observed in Table 1.

Among these proteins, we focused on tubulins and their isoforms. Table 2 provides a comparison of the tubulins identified in the K562 and Lucena 1 groups. Out of 24 identified isoforms, 19 are present in the Lucena 1 group, and 21 isoforms are in the K562 group. Notably, the isoform Gamma-tubulin complex component 6, Tubulin beta 8B, and Tubulin alpha chain-like 3 are exclusive to the Lucena 1 group.

**Figure 1:**
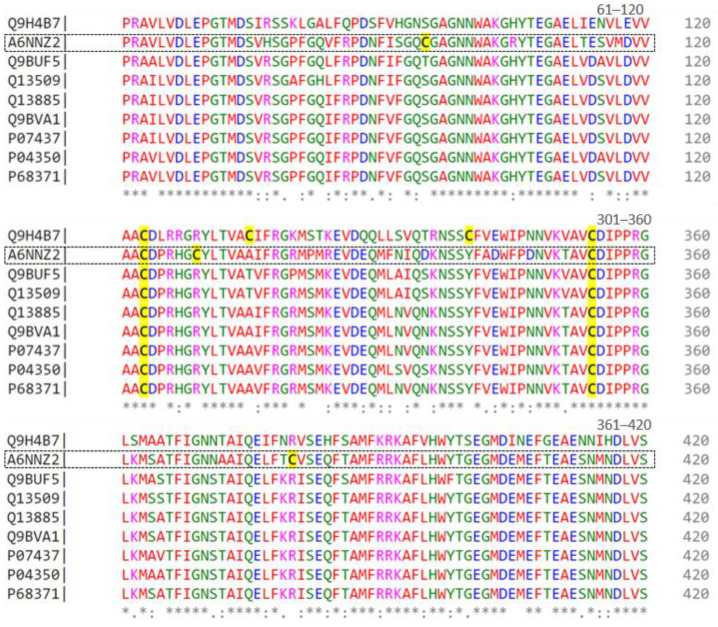
Alignment of beta-tubulin isoforms (from residues 61 to 420) found in the proteome of the Lucena 1 cell line by CLUSTAL O (1.2.4) multiple sequence alignment. Conserved residues are indicated by asterisks (*), while dots (.) denote residue substitutions.

To further analyze these tubulins, Figure 1 displays an alignment of nine sequences represented by UNIPROT codes. Residues 61 to 420 of each sequence are depicted, with colors indicating their chemical properties, such as charge, polarity, or hydrophobicity. The symbols below the residues denote the degree of similarity, where ‘*’ signifies identical residues, ‘:’ indicates very similar residues, and ‘.’ denotes slightly similar residues. Numbers on the right side indicate the position of residues in each sequence, with cysteines highlighted in yellow.

**Figure 2:**
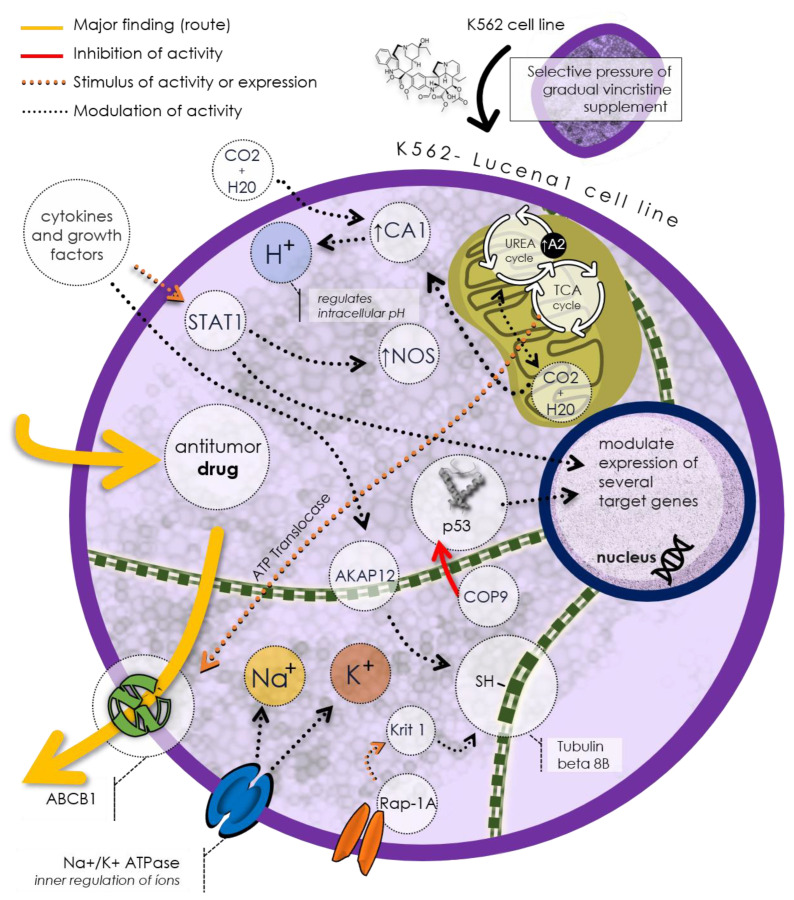
The Lucena 1 cell line proposal of a distinctive phenotype implicated in drug resistance, notably expressing P-gp/ABCB1, a prominent efflux pump responsible for extruding antitumor drugs. Regulatory control over intracellular pH is mantained by TCAI, a carbonic anhydrase isoform, while Na^+^/K^+^ ATPase preserves ion gradients. Lucena 1 modulates various genes, affecting processes like the urea cycle, citric acid cycle, apoptosis, and cell signaling. These mechanisms influence Lucena 1 cell responses to antitumor drugs, ultimately affecting their resistance or sensitivity.

Figure 2 illustrates a potential mechanism of drug resistance in the Lucena 1 cell line based on the discovered unique proteins. As mentioned before, Lucena 1 cells express the P-gp/ABCB1, capable of expelling antitumor drugs from the cell, thus reducing their efficacy. This protein exemplifies an efflux pump, a common drug resistance mechanism in cancer cells. Lucena 1 cells also regulate the intracellular pH using TCAI, an isoform of carbonic anhydrase, to control hydrogen ion (H^+^) balance in the cytoplasm. Additionally, Lucena 1 cells utilize Na^+^/K^+^ ATPase, an ion pump maintaining sodium (Na^+^) and potassium (K^+^) gradients across the cell membrane. Lucena 1 modulates the expression of various target proteins involved in processes such as the urea cycle, citric acid cycle (TCA), cell cycle regulation (COP9), cell adhesion (Krit1), cell signaling (Rap-1A), and cytoskeletal formation (tubulin beta 8B and tubulin alpha chain-like 3). These proteins may influence the Lucena 1 cell response to antitumor drugs, rendering it more resistant or less sensitive.

## 4. Discussion

The Lucena 1 cell line is a MDR descendant from the CML cell line K562 by the selective pressure of gradual supplementation with vincristine [9]. In order to further contribute to describing the protein pattern related to this MDR cell line, we performed a proteomic analysis comparison between K562 and Lucena 1 cell lines.

The results suggested that the phenotypic characteristics of the Lucena 1 cell line are qualitative and not quantitative. The label-free analysis did not show significant results between cell lines; however, the unique proteins revealed (Table 1) reaffirm the already existing descriptive literature and add information that solves intrinsic characteristics, such as drug resistance.

The main finding of these data is the protein Translocase ABCB1 (P08183), which is an ATP-dependent efflux pump responsible for eliminating substances, including chemotherapeutic drugs, in multiresistant cells [18,19]. In order to understand the distinctive metabolic dynamics of this cell and the emergence of this protein defining it as MDR, we revisit its selection by vincristine. Vincristine, an alkaloid chemotherapeutic agent employed in treating diverse cancer forms, acts by binding to tubulin or microtubules [20]. This binding mechanism inhibits the polymerization of mitotic spindle microtubules, leading to structural damage and preventing cell mitosis [21,22]. Its correlations encompass interactions with various proteins, including those involved in energy metabolism (such as ATP/ADP translocase 1) and signaling proteins (such as signal transducer and activator of transcription 1-alpha/beta), influencing the cellular environment. Additionally, ABCB1 may have indirect interactions with metabolic enzymes, affecting drug metabolism by cytochrome P450 enzymes [20]. Its involvement with transporters and its role in regulating the intracellular balance of substrates, such as ions and organic molecules, further underscore its significance. In interactions with signaling proteins, ABCB1 can impact cellular responses to external stimuli and modulate intracellular signaling [23].

Considering the role of vincristine in the formation of the mitotic spindle, we carried out the analysis of tubulins (Table 2) to verify if there were any discrepancies between the cell lines. We observed that most tubulin isoforms are shared among cell lines, except for Tubulin beta 8B (A6NNZ2) and Tubulin alpha chain-like 3 (A6NHL2), a protein exclusive to the Lucena 1. Compared to other beta-tubulins, this isoform has three additional cysteines in its sequence, at positions 95, 309, and 380 (Figure 1). This characteristic could lead to a distinct conformation, potentially ensuring protection against the effects of vincristine and/or facilitating cellular restructuring. Nevertheless, this hypothesis requires experimental validation.

Relative to cytoskeleton, we also found an association between Ras-related protein Rap-1A (Rap1A) and Krev1 interaction trapped gene 1 (Krit1) (Supplementary Table S1). Krit1 can inhibit microtubule polymerization, resulting in decreased microtubule stability and reduced cell dynamics. It is important to note that Krit1 is regulated by several factors, including calcium concentration and the presence of cellular stressors [24]. The cytoskeleton remains intricately involved in the intracellular transport of vesicles and organelles, facilitating the efficient incorporation of drug-resistant proteins, such as ABC family glycoproteins, into the cell membrane. Moreover, it influences the subcellular distribution of membrane transporters, like P-gp, directly impacting their efficacy in drug expulsion. Morphological changes, often mediated by the cytoskeleton, play a crucial role. Some alterations were already observed by [9].

Among the key findings is the involvement of the signal transducer and activator of transcription 1-alpha/beta (STAT1) protein in regulating gene expression triggered by specific signaling pathways, notably those associated with cellular stress and inflammation [25]. STAT1 is activated by phosphorylation in response to signals such as interferon-gamma (IFN-γ) and may play a role in regulating the expression of genes involved in the urea cycle [25]. Notably, one such gene is associated with the protein Arginase 2 (A2), an enzyme that catalyzes the hydrolysis of arginine, a precursor of nitric oxide (NO), as confirmed through distinctive protein analysis (Table 1) [26]. Although STAT1 has already been associated with a tumor suppressor role, typically linked to IFN-γ signaling [27,28], Kovacic (2006) [29] proposes that STAT1 acts as a tumor promoter in the development of leukemia. Cells lacking expression of this gene showed increased MHC class I expression following leukemia progression, potentially offering benefits to leukemia patients.

Still analyzing the urea cycle, it is noteworthy to mention the increase in the protein carbonic anhydrase I (CAI). This cytosolic isoform of carbonic anhydrase is found in various tissues throughout the body, such as erythrocytes, kidneys, and pancreas. Although CAI is not directly involved in the urea cycle, it does contribute to intracellular pH regulation by catalyzing the reversible hydration of CO_2_ to form HCO_3_^−^ and H^+^, thereby releasing protons into the intracellular milieu [30].

One notable protein is AKR1, which encompasses a range of proteins involved in carbohydrate metabolism, cellular signaling, and antioxidant defense processes. Certain AKR1 isoforms have been linked to pathological processes, including cancer. In some instances, it has been found to be overexpressed in cancer cells and associated with drug resistance and chemoresistance [31].

Another feature found in the Lucena 1 cell line was the COP9 signalosome complex subunit 5 (COP9). COP9 targets the human p53 and has the characteristic of being a tumor suppressor protein [32]. The p53 is degraded by the ubiquitin-26S proteasome system, and phosphorylation of the COP9 signalosome targets p53 for ubiquitin-26S proteasome-dependent degradation [33].

In summary, one of the most relevant descriptions is ABCB1 protein, also known as P-gp, a transmembrane protein. While certain drugs have been designed to inhibit ABCB1 activity, aiming to enhance the effectiveness of cancer therapies, such as the calcium channel blocker verapamil, a major concern arises from the expression of these transporters also in normal cells, which may result in unacceptable toxicity [34,35].

Therefore, unraveling new proteins associated with the MDR mechanism may be useful for innovating cancer models and antitumor targeted strategies.

## 5. Conclusions

In summary, the proteomic analysis revealed qualitative distinctions in the phenotypic characteristics of the Lucena 1 cell line, underscoring the significance of qualitative over quantitative variations. Particularly noteworthy was the identification of Translocase ABCB1 as a pivotal player. This protein operates as an ATP-dependent efflux pump, responsible for drug elimination in MDR cells, thereby highlighting its significance in the MDR phenotype. The exclusive presence of the unique tubulin isoforms, Tubulin beta 8B, in Lucena 1, presents intriguing possibilities regarding its role in protecting against vincristine actions or contributing to cellular restructuring. Additionally, the role of Rap-1A and Krit1 in cytoskeletal regulation underscored the complex interplay that influences both microtubule stability and cellular dynamics.

The presence of STAT1, recognized for its involvement in cellular stress and inflammation responses, added another layer of complexity, with contrasting views on its role as a tumor suppressor or promoter in leukemia development. The increased expression of carbonic anhydrase I and the discovery of COP9 further enriched our understanding of Lucena 1’s unique molecular profile. Moreover, the identification of novel proteins, including COP9, in Lucena 1 suggests potential applications in the development of new cancer models and antitumor strategies. Overall, our study contributes valuable insights into the field, paving the way for continued exploration and innovation in cancer research and treatment.

## Figures and Tables

**Table 1:** Table of unique proteins, with selected proteins with −10lgP > 90, identified in proteomic analysis for the Lucena 1 cell line.

Accession	−10lgP	Peptides	Unique	Description
A6NNZ2	163.21	22	0	Tubulin beta 8B
P00915	158.22	17	15	Carbonic anhydrase 1
Q58FF8	153.51	24	0	Putative heat shock protein HSP 90-beta 2
P63267	147.82	19	1	Actin, gamma-enteric smooth muscle
P62736	147.82	19	1	Actin, aortic smooth muscle
P08183	147.56	29	23	ATP-dependent translocase ABCB1
Q8N257	130.83	18	1	Histone H2B type 3-B
Q02952	126.10	13	13	A-kinase anchor protein 12
P09960	125.99	19	18	Leukotriene A-4 hydrolase
Q6FI13	124.98	11	1	Histone H2A type 2-A
Q16777	124.98	11	1	Histone H2A type 2-C
P13929	123.72	13	1	Beta-enolase
P62140	121.09	11	1	Serine/threonine-protein phosphatase PP1-beta catalytic subunit
P42330	119.37	16	2	Aldo-keto reductase family 1 member C3
Q92597	116.56	7	6	Protein NDRG1
P0DMV1	114.95	10	9	Cancer/testis antigen family 45 member A8
P0DMV2	114.95	10	9	Cancer/testis antigen family 45 member A9
Q5DJT8	114.95	10	9	Cancer/testis antigen family 45 member A2
P42224	114.80	11	10	Signal transducer and activator of transcription 1-alpha/beta
P52895	114.13	20	2	Aldo-keto reductase family 1 member C2
Q32MZ4	112.01	10	7	Leucine-rich repeat flightless-interacting protein 1
Q96L21	110.23	13	1	60S ribosomal protein L10-like
Q9BYX7	110.10	8	0	Putative beta-actin-like protein 3
P67775	107.30	11	10	Serine/threonine-protein phosphatase 2A catalytic subunit alpha isoform
P48741	106.13	10	1	Putative heat shock 70 kDa protein 7
P29966	106.09	7	7	Myristoylated alanine-rich C-kinase substrate
Q92905	104.65	8	8	COP9 signalosome complex subunit 5
P12235	102.26	12	1	ADP/ATP translocase 1
P68871	100.08	11	3	Hemoglobin subunit beta
P13637	95.17	11	1	Sodium/potassium-transporting ATPase subunit alpha-3
P00918	94.77	7	6	Carbonic anhydrase 2
Q92526	94.17	9	1	T-complex protein 1 subunit zeta-2
P78540	93.28	6	6	Arginase-2, mitochondrial
P35609	93.05	9	0	Alpha-actinin-2
P11310	92.23	8	7	Medium-chain specific acyl-CoA dehydrogenase, mitochondrial
P62834	92.19	10	2	Ras-related protein Rap-1A

**Table 2:** Comparative proteomic analysis of tubulins found in K562 and Lucena 1 cell lines.

Accession	Description	K562			Lucena 1		
		−10lgP	Peptides	Unique	−10lgP	Peptides	Unique
Q9BSJ2	Gamma-tubulin complex component 2	39.48	4	0	-		
Q96RT7	Gamma-tubulin complex component 6	-			31.55	3	0
Q5TCY1	Tau-tubulin kinase 1	35.75	3	0	-		
Q71U36	Tubulin alpha-1A chain	218.38	36	0	184.98	36	0
P68363	Tubulin alpha-1B chain	220.29	37	0	185.86	36	0
Q9BQE3	Tubulin alpha-1C chain	213.37	36	4	187.45	38	5
P68366	Tubulin alpha-4A chain	204.77	32	2	174.66	28	0
Q9NY65	Tubulin alpha-8 chain	173.82	20	0	157.81	19	1
A6NHL2	Tubulin alpha chain-like 3	-			73.80	6	0
P07437	Tubulin beta chain	236.39	37	5	207.07	45	4
Q9H4B7	Tubulin beta-1 chain	88.50	7	0	95.06	9	0
Q13885	Tubulin beta-2A chain	210.40	30	1	189.20	39	1
Q9BVA1	Tubulin beta-2B chain	211.00	31	1	189.08	40	1
Q13509	Tubulin beta-3 chain	177.54	20	0	168.44	27	0
P04350	Tubulin beta-4A chain	224.07	32	0	-		
P68371	Tubulin beta-4B chain	232.14	36	1	203.80	45	3
Q9BUF5	Tubulin beta-6 chain	179.35	26	7	163.04	29	6
A6NNZ2	Tubulin beta 8B	-			163.21	22	0
Q14679	Tubulin monoglutamylase TTLL4	30.73	3	0	31.13	2	0
Q6EMB2	Tubulin polyglutamylase TTLL5	26.06	2	0	-		
Q99426	Tubulin-folding cofactor B	37.43	2	2	-		
O75347	Tubulin-specific chaperone A	97.98	8	7	106.78	9	9
Q9BTW9	Tubulin-specific chaperone D	51.27	3	1	66.97	6	2
Q14166	Tubulin--tyrosine ligase-like protein 12	97.81	9	7	106.83	14	10

## Data Availability

Raw data of mass spectrometry analysis is available at https://repository.jpostdb.org/ (accessed on 13 August 2024) entry JPST003033 PXD053987 (https://proteomecentral.proteomexchange.org/cgi/GetDataset?ID=PXD053987 accessed on 13 August 2024).

## Supplementary Materials

The following supporting information can be downloaded at: https://www.mdpi.com/article/10.3390/cells13171427/s1, Supplementary Table S1: Table of proteins, identified in proteomic analysis for the Lucena 1 cell line, with −10lgP > 40.
